# Plasmon Hybridizations in Compound Nanorod–Nanohole Arrays

**DOI:** 10.3390/nano13142135

**Published:** 2023-07-23

**Authors:** Shahab Razavi, Yiping Zhao

**Affiliations:** Department of Physics and Astronomy, University of Georgia, Athens, GA 30602, USA; shahab.razavi@gmail.com

**Keywords:** enhanced optical transmission, plasmon hybridization, nanohole arrays, nanorod

## Abstract

This study shows that a hybridized plasmonic mode, represented by an additional transmission peak, in a compound structure consisting of a nanorod embedded in a nanohole can be effectively described as a quasi-dipole oscillator. When two nanorods are introduced into a nanohole, these two quasi-dipoles can couple and hybridize, giving rise to two additional transmission peaks in the enhanced optical transmission spectrum. The relative intensities of these peaks can be controlled by adjusting the incident polarization, while their separations can be tuned by modifying the length of the nanorods. The concept of quasi-dipoles in compound nanohole structures can be further extended to predict the coupling behavior of even more complex compound configurations, such as multiple nanorods within nanoholes, resulting in the generation of multiple hybridization states. Consequently, the shape and response of the transmission peaks can be precisely engineered. This strategy could be used to design nanohole-based metasurfaces for applications such as ultra-thin optical filters, waveplates, polarizers, etc.

## 1. Introduction

The discovery of enhanced optical transmission (EOT) through subwavelength hole arrays in 1998 has attracted a great deal of research effort in both fundamental understanding and practical applications [1,2,3]. EOT is primarily influenced by surface plasmon polaritons (SPPs) and localized surface plasmons (LSPs). The shape, size, and spacing of the holes, as well as the incident light configurations, play crucial roles in determining the properties of EOT [2,3].

Recently, compound nanohole structures with discrete plasmonic elements inside the nanoholes have emerged as a fascinating area of study. These structures have been found to dramatically alter the wavelength response and shape of EOT peaks, introduce Fabry–Perot-like guiding modes, enhance Fano resonance, and generate peak splitting and strong localized electric fields [4,5,6,7]. Notably, annular aperture arrays (AAAs) introduced by Baida and Van Labeke have garnered significant attention [7]. Thin AAAs can shift the resonant wavelength of EOT, create high electric fields in the nanodisk-nanohole gaps [8,9,10], improve the performance of EOT-based sensors [11,12,13], serve as substrates for surface-enhanced infrared absorption spectroscopy [14] and surface-enhanced Raman scattering [15], and even be utilized for constructing waveplates [16], etc. Additionally, nanorod-in-nanohole (NR-in-NH) structures have been extensively investigated both theoretically [17,18,19] and experimentally [20,21]. It has been observed that multiple parameters, such as hole diameter, rod length and aspect ratio, rod position, and the period of the nanohole array, can be simultaneously tuned to control the plasmonic properties, enabling multiple instances of coupling between SPPs and LSPs.

Many phenomena observed in AAAs and NR-in-NH structures can be explained using hybridization models proposed by Prodan et al. [22]. For example, Yani et al. demonstrated that rectangular coaxial cavity arrays generate two distinct EOT peaks due to the hybridization between the dipole resonance of the nanorods inside the cavity and the LSP resonance of the rectangular cavity [23]. The hybridization model treats each discrete plasmonic structure as a virtual plasmonic atom. In the case of AAA structures, the nanohole and the central disk can be considered as two separated plasmonic atoms. Similarly, in NR-in-NH structures, the nanorod and the nanohole can be treated as two plasmonic atoms. However, due to the strong LSP property exhibited by NRs and the presence of both SPP and LSP properties in NH arrays, the optical response of NR-in-NH structures not only incorporates the SPP and LSP properties of the nanohole array, which result in EOT features, but also gives rise to a new EOT peak at a significantly longer wavelength than the LSP wavelength of the NRs [17,20,24]. This new peak, or plasmonic mode, emerges as a consequence of the coupling or hybridization between two relatively simple plasmon modes or “atoms”, namely the NR and NH. An intriguing question arises: can this new EOT mode, or the hybridized mode, be regarded as another plasmonic atom capable of hybridizing with similar structures? For instance, if multiple nanorods are present inside a nanohole, can each NR-in-NH structure be treated as a distinct plasmonic atom, and would hybridization occur among all these atoms? And how would such extensive hybridization affect the optical properties of these structures? In essence, can we employ a straightforward physical analogy to describe the resulting plasmon mode of compound NR-in-NH structures, even in more intricate compound structures? Exploring the possibilities of hybridization among compound NR-in-NH structures promises to uncover new and exciting plasmonic phenomena.

In this paper, we present a novel finding that expands upon these previous investigations. We observe that the hybridized plasmonic mode in NR-in-NH structures can be described as a quasi-dipole oscillator, and the addition of new nanorods into a nanohole can be treated as the hybridization of the quasi-dipoles. This additional hybridization process introduces extra EOT modes in the transmission spectrum and significantly extends the tunability of the EOT properties of compound NR-in-NH structures. Our findings shed new light on the intricate plasmonic behavior of compound nanohole structures and provide valuable insights for designing and engineering tunable optical devices with enhanced performance.

## 2. Materials and Methods

**Figure 1:**
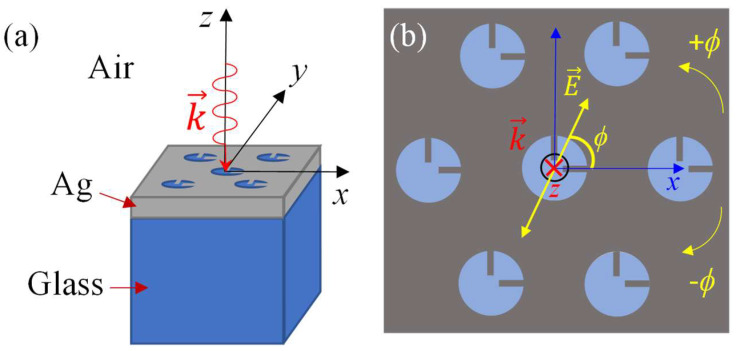
(**a**) The schematics of the 2RNH structure, light incident configuration, and coordinates. (**b**) The top view of the 2RNH structure and the definition of the polarization angle *ϕ* with respect to the *x*-axis. Here, $\vec{k}$ represents the wave vector.

The proposed structure in this study is a two-rods-in-a-nanohole (2RNH) metasurface positioned on a glass substrate, as depicted in Figure 1. The nanoholes, with a radius of *r* = 170 nm, are arranged in a hexagonal pattern with a lattice constant of 500 nm (Figure 1a). Each nanohole contains two identical rods oriented perpendicular to each other (Figure 1b). The width of the rods is kept constant at *w* = 20 nm while the length *l* varies from 17 nm to 157 nm, in order to investigate the impact of rod length on the plasmonic behavior. The thickness of the nanorods and the Ag film is 90 nm (the grey shaded area of Figure 1b). 

Numerical simulations were performed using commercially available finite-difference time-domain (FDTD) software, specifically FDTD Solutions v 8.19.1584, Lumerical Solutions Inc. The simulation unit cell was defined as a rectangular prism with dimensions of 0.5 × 0.866 × 3.0 μm^3^ along the *x*, *y*, and *z* directions, respectively. A plane wave was incident perpendicularly (towards the negative *z*-direction) onto the structure with a polarization angle *ϕ*, where *ϕ* ranged from −90° to +90° with respect to the *x*-axis, as illustrated in Figure 1b. Perfectly matched layer (PML) boundary conditions were applied to the top and bottom surfaces of the calculation domain. The mesh size was set to 2.5 nm along all three axes. Two monitors, “frequency-domain field profile” and “frequency-domain field and power”, with a size of 5 × 5 μm^2^, were placed directly above the silver layer at the air-silver interface to record the electric field distribution and transmission spectra. The material properties, specifically permittivity and permeability, of the glass and silver layers were obtained from ref. [25].

## 3. Results and Discussion

**Figure 2:**
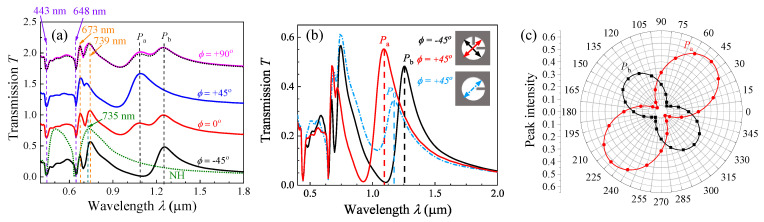
(**a**) Polarization dependence *T*(*λ*) for a 2RNH structure with *l* = 102 nm. Different colored spectrum corresponds to the same colored *ϕ* value in the figure. The green dashed curve is the calculated *T*(*λ*) for the NH arrays of the same *D* and *r*. In order to enhance the clarity of the spectra, they have been vertically shifted by a constant transmission value. (**b**) Comparison of $T_{1}(\lambda )$ of the 1RNH structure (at *ϕ* = +45°) to $T_{+45}\left(\lambda \right)$ and $T_{-45}\left(\lambda \right)$ of the 2RNH structure for *l* = 153 nm. The peak *P*_0_ of the 1RNH structure is located between the *P*_a_ and *P*_b_ peaks, and (**c**) the polar plot of the transmission intensity *T*(*λ*_a_) and *T*(*λ*_b_) versus *ϕ* is shown. The solid curves are the fittings according to the cos^2^ rule.

Figure 2a shows representative transmission spectra *T*(*λ*) for *l* = 102 nm (the original spectra are shown in Figure S1 of the Supplementary Materials). To compare, we also calculated the *T*(*λ*) for nanohole (NH) arrays with the same *D* and *r* (the green dashed curve in Figure 2a). When *λ* < 1000 nm, the *ϕ*-dependent spectra show similar features and are consistent with the NH spectrum, except that there is a peak splitting at around *λ* = 735 nm (the EOT peak at the Ag-glass interface for NH). All the spectra have two valleys at *λ* = 443 nm and 648 nm, which correspond to Wood’s anomaly [26]. The broad transmission peak from 648 nm to 1000 nm is due to EOT for the NH arrays. However, for the spectra of 2RNH at different *ϕ*, the broad EOT peak originally at *λ* = 735 nm in the NH arrays splits into two peaks, one at 670–678 nm and the other at 720–746 nm. Such a peak splitting is due to the hybridization of the SPP resonance of NHs and LSP resonance of nanorods [23]. In addition to these common spectral features, two extra peaks, *P*_a_ and *P*_b_, appear around the near infrared wavelength range: one at *λ*_a_ = 1085 nm and the other at *λ*_b_ = 1266 nm. For *ϕ* = ±45°, only one of the two peaks appears, while for *ϕ* ≠ ±45°, both the peaks appear in the spectra with different relative intensities. In fact, if we choose the spectra (*T*_±45_(*λ*)) at *ϕ* = ±45° as two bases (or eigenstates), then the transmission spectrum $T_{\phi }\left(\lambda \right)$ at any arbitrary polarization angle *ϕ* can be expressed as

(1) $$T_{\phi }\left(\lambda \right)=T_{+45}\left(\lambda \right){sin}^{2}\left(\phi -45{}^{\circ}\right)+T_{-45}\left(\lambda \right){cos}^{2}\left(\phi +45{}^{\circ}\right)$$

The black dotted curve in Figure 2a is such a fitting for the *ϕ* = 90° spectrum using Equation (1). Despite some small differences, the experimentally measured $T_{90}\left(\lambda \right)$ matches very well with the spectrum obtained from Equation (1). This result shows that the two spectra at *ϕ* = ±45°, *T*_+45_(*λ*) and *T*_−45_(*λ*), can be used as orthogonal bases to construct other transmission spectra. If the *λ*_a_ = 1085 nm peak at *ϕ* = +45° and the *λ*_b_ = 1266 nm peak at *ϕ* = −45° are considered new plasmonic modes of the 2RNH structures, then these two modes should also be the eigenmodes. 

To understand how the two extra transmission modes occur, we calculated the polarization-dependent *T*_1_(*λ*) of the one-rod-in-a-nanohole (1RNH) structure and compared the spectrum with the *T*_+45_(*λ*) and *T*_−45_(*λ*) of the 2RNH for *l* = 153 nm. Figure 2b shows the *T*_1_(*λ*) at *ϕ* = +45° for *l* = 153 nm. In addition to a peak split at *λ* = 735 nm (compared to the NH spectra shown in Figure 2a), the *T*_1_(*λ*) shows an additional transmission peak *P*_0_ at *λ*_0_ = 1172 nm. Once *l* is fixed, the location of *λ*_0_ remains unchanged except that when *ϕ* = 0°, the *P*_0_ peak disappears. This implies that the plasmonic mode at *λ*_0_ = 1172 nm is only excited when the polarization of the incident light is parallel to the nanorod axis [17]. This observation is consistent with previous reports on similar structures [27]. Comparing the *T*_+45_(*λ*) and *T*_−45_(*λ*) of the 2RNH structure, we note that *λ*_a_ < *λ*_0_ < *λ*_b_. The peak transmissions at *λ*_a_ and *λ*_b_ depend on the polarization angle *ϕ*. Figure 2c presents the polar plots of both the *T*(*λ*_a_) and *T*(*λ*_b_) functions of *ϕ* based on Figure 2b. Both *T*(*λ*_a_) and *T*(*λ*_b_) versus *ϕ* follow a dumbbell shape, with their long axes precisely perpendicular to each other. We can fit each curve using the following function:

(2) $$T\left(\lambda_{a,b}\right)=T_{a.b}\cos^{2}\left[2\left(\phi \mp 45{}^{\circ}\right)\right],$$

where $T_{a}$ and $T_{b}$ represent the maximum transmission at *λ*_a_ and *λ*_b_, respectively. The “−” sign is used for *T*(*λ*_a_), while the “+“ sign is used for *T*(*λ*_b_). The solid curves in Figure 2c show the fitting results, yielding $T_{a}=0.57$ and $T_{b}=0.37$, respectively. This analysis further confirms that both the *λ*_a_ peak at *ϕ* = +45° and the *λ*_b_ peak at *ϕ* = −45° can be considered as two new plasmonic modes of the 2RNH structures, or they could be interpreted as two new eigenmodes resulting from the coupling within the 2RNH structure.

**Figure 3:**
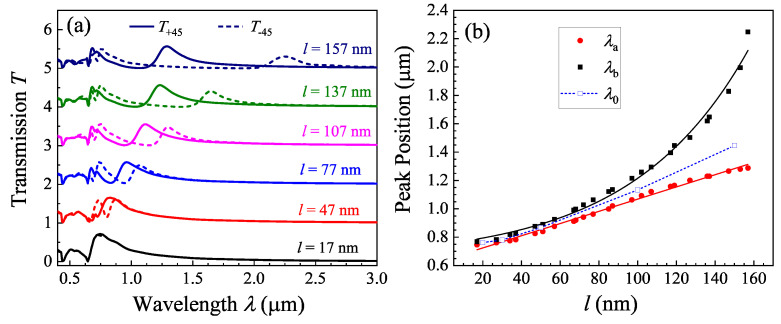
(**a**) The $T_{+45}\left(\lambda \right)$ (solid curves) and $T_{-45}\left(\lambda \right)$ (dashed curves) for a 2RNH structure with different *l*. They show that both *λ*_a_ and *λ*_b_ redshift with *l*. In order to enhance the clarity of the spectra, they have been vertically shifted by a constant transmission value. (**b**) The plots of the extracted *λ*_0_, *λ*_a_, *λ*_b_, and *λ*_b_ − *λ*_a_ from FDTD spectra versus *l*. The plot for *λ*_b_ − *λ*_a_ versus *l* is in semi-log scale. The *λ*_0_ and *λ*_a_ follow a linear relationship with *l*, while the *λ_b_* follows a power law (see the solid and dashed curves from the fitting results).

Such a phenomenon is not only observed for *l* = 102 and 153 nm structures, but also for other *l*. Figure 3a shows the *T*_+45_(*λ*) and *T*_−45_(*λ*) spectra for different *l* (the original spectra are shown in Figure S2 of the Supplementary Materials). When *l* is small (=17 nm), the *T*_+45_(*λ*) and *T*_−45_(*λ*) almost overlap with each other, and the spectra are very similar to those of the NH arrays shown in Figure 2a. However, when *l* ≥ 47 nm, two peaks start to appear: the shorter-wavelength peak *λ*_a_ is in *T*_+45_(*λ*) and the other, *λ*_b_, in *T*_−45_(*λ*). With the increase in *l*, both *λ*_a_ and *λ*_b_ redshift, and the separation between *λ*_a_ and *λ*_b_ becomes larger and larger. Figure 3b plots the *λ*_a_, *λ*_b_, and *λ*_b_−*λ*_a_ versus *l*. The *λ*_a_ increases almost linearly with *l* and can be fit by a linear function ($\lambda_{a}=4.3l+638$ nm), while the *λ*_b_ raises with *l* in an exponential fashion ($\lambda_{b}=109e^{l/60}+642$ nm). Since the two fittings have similar intersections, the 60 nm in the exponent characterizes a critical rod length above which the two modes *P*_a_ and *P*_b_ start to separate rapidly. We also calculated other 1RNH structures with different *l* and plotted *λ*_0_ versus *l* in Figure 3b. The *λ*_0_ versus *l* follows a linear relationship. However, regardless of the rod length *l*, one observes that the relationship of *λ*_a_ < *λ*_0_ < *λ*_b_ is always true.

Based on our recent experimental and numerical calculation for tilted Ag nanorods on Ag NH arrays, the appearance of the additional *λ*_0_ transmission peak in the 1RNH structure is due to a dipole-oscillation-induced enhanced transmission from the nanorods [17,18,19,20,24]. Similar understanding can be applied to the 1RNH. The addition of an extra-nanorod in the perpendicular position could also introduce a dipole-like oscillation. This second dipole oscillation could couple with the dipole oscillation of the existing nanorod when excited by different polarization incidence, shift the resonance wavelength to red or blue, and generate hybridized plasmon modes.

**Figure 4:**
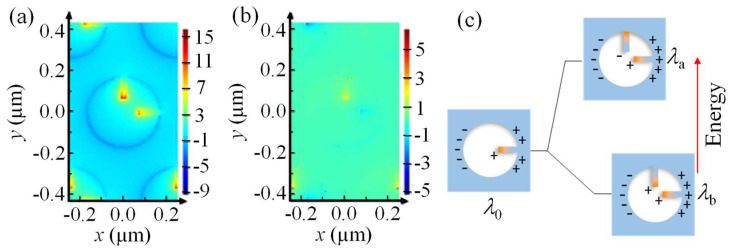
The *z* component of local electric field distribution $\frac{E_{z}}{E_{0}}$ at the Ag-air interface of the *l* = 107 nm 2RNH structure for (**a**) *λ_a_* at *ϕ* = +45° and (**b**) *λ_b_* at *ϕ* = −45°. (**c**) The illustration of the hybridization model of the dipole oscillations of two nanorods in a 2RNH structure.

To better understand the two modes at *λ*_a_ and *λ*_b_, the z component local electric field maps of the Ag-air interface for the 2RNH structure (*l* = 107 nm) at *λ*_a_ and *λ*_b_ are obtained, as shown in Figure 4. Figure 4a shows the $\frac{E_{z}}{E_{0}}$ map for *λ*_a_ at *ϕ* = +45°. Strong dipole-like field distributions are seen in both nanorods; the vertical nanorod has a high local field in the bottom, indicating an oscillation from bottom to top, while the horizontal nanorod has a high field at the left end, inferring an oscillation direction from left to right. If the oscillation field directions of the two nanorods are projected to the *ϕ* = +45° direction (polarization direction), they are in-phase and parallel to each other. This is equivalent to a nanorod that is placed in the *ϕ* = +45° direction but its effective dipole oscillation length is smaller compared to *l*. Thus, a smaller resonant wavelength (*λ*_a_) is expected, and it is expected that the resonant wavelength *λ*_a_ is proportional to *l*. Figure 4b shows the $\frac{E_{z}}{E_{0}}$ field map for *λ*_b_ at *ϕ* = −45°. One can still observe two dipole-like oscillations, but they have a head-to-tail arrangement. The vertical nanorod has a high local field in the bottom, showing an oscillation from bottom to top; while the horizontal rod has a low field at the left end, inferring an oscillation from right to left. If the oscillation directions of the two apparent dipoles are projected to the *ϕ* = −45° direction, they are in-phase and in series with each other, which infers that the effective dipole oscillation length is larger compared to *l*, and one should expect a larger resonant wavelength (*λ*_b_). Hence, the newly observed plasmonic mode *P*_0_ in Figure 2b of the 1RNH structures can be effectively treated as a quasi-dipole. Its emergence is a result of the coupling between the NR and NH. In the case of the 2RNH structures, the two 1RNH configurations in the horizontal and vertical directions can be considered as two coupled quasi-dipoles, and the formation of two new eigenmodes, *P*_a_ and *P*_b_, can be described as a hybridization of these coupled quasi-dipoles, as shown in Figure 4c. When the two quasi-dipoles oscillate in parallel, an anti-bonding state is realized with higher emission energy. Conversely, when the quasi-dipoles oscillate in series, a bonding state is achieved, resulting in lower emission energy. In the case of the quasi-dipoles arranged in series (*ϕ* = −45° case), the coupling between them is expected to be stronger compared to the *ϕ* = +45° case. This hybridization framework can also explain the *λ*_a_ − *l* and *λ*_b_ − *l* relationships shown in Figure 3b: as the NR length *l* increases, the coupling between the two quasi-dipoles strengthens due to the shorter distance between the tips of the nanorods. As a result, the separation between *λ*_a_ and *λ*_b_ increases, following a nonlinear relationship [28,29,30].

**Figure 5:**
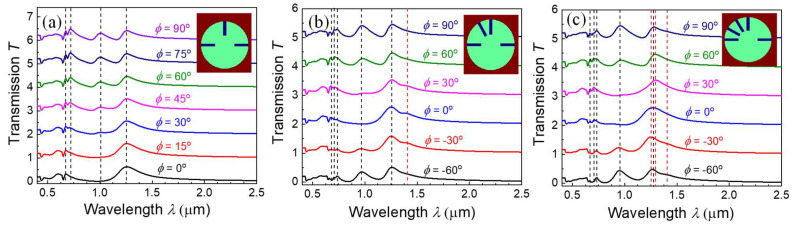
The polarization-dependent *T*(*λ*) spectra for (**a**) a 3-nanorod structure, (**b**) a 4-nanorod structure, and (**c**) a 5-nanorod structure. The dashed lines indicate the peak positions that appeared in *T*(*λ*) spectra at different *ϕ.* The spectra in (**a**) are similar to those of Figure 2a, while the addition of nanorods on the non-orthogonal directions generates new resonance peaks in (**b**,**c**). In order to enhance the clarity of the spectra, they have been vertically shifted by a constant transmission value.

Since the nanorods inside a nanohole can be treated as a quasi-dipole and they can couple differently based on incident polarization, it is expected that multiple modes could be generated if more nanorods appears in a nanohole, i.e., each nanorod could act as a plasmonic “atom”, and one expects that new transmission peaks generated by multiple hybridizations could shift or change the entire transmission spectrum. Figure 5 shows the polarization-dependent *T*(*λ*) spectra of three, four, and five nanorods inside a nanohole for *l* = 102 nm (the original spectra are shown in Figure S3 of the Supplementary Materials), and the nanorod configurations are illustrated as the inserts. When a new nanorod is added to another horizontal direction of 2RNH (Figure 5a), the polarization-dependent *T*(*λ*) spectra show no additional peak, and only the *λ*_a_ (=1085 nm) and *λ*_b_ (=1266 nm) peaks remain. When another nanorod is added at 120°, as shown in Figure 5b, the *λ*_a_ peak is relocated at 967 nm and the *λ*_b_ peak changes to 1257 nm, and both are slightly blueshifted. However, an additional shoulder occurs at *λ*_c_ = 1406 nm at *ϕ* = 0° and *ϕ* = ±30°, as indicated by the vertical red dashed line. This mode is due to additional dipole-like hybridization in multi-nanorod configurations. When a fifth nanorod is added at 150°, as shown in Figure 5c, at least three more peaks at $\lambda_{c}^{\prime }$, $\lambda_{c}^{\prime \prime }$, and *λ*_c_ = 1233, 1295, and 1404 nm, respectively, can be identified in addition to the *λ*_a_ = 946 nm and *λ*_b_ = 1262 nm peaks. These new modes are due to the complicated dipole-like coupling in multi-nanorod arrangement in a hole structure. One can use more nanorods or different nanorod lengths to tune the hybridization among the dipoles and design the spectral range and width of the transmission peaks, which could be used for ultra-thin optical filters, waveplates, absorbers, etc. Theoretically, one could imagine that if more and more nanorods are added, a bandgap-like structure could be formed.

## 4. Conclusions

In summary, we have shown that the new mode generated by a compound 1RNH structure due to the coupling between the NR and NH can be treated as a quasi-dipole, and in a 2RNH compound structure, the two nanorods can act like coupled quasi-dipole oscillators and introduce additional transmission peaks in the traditional EOT spectrum of NH arrays. When two nanorods in the hole are arranged perpendicular to each other, the quasi-dipoles could couple differently depending on the incident polarization. When the two dipoles oscillate parallel to each other, an anti-bonding state is realized; if the two dipoles oscillate in series, a bonding state is achieved. Such a coupling leads to a splitting in the transmission peak of the new plasmonic mode observed in the 1RNH structure. Based on such a coupling strategy, multiple nanorods can be introduced into the nanoholes and generate multiple coupling effects so that multiple new transmission peaks can be induced or the transmission peaks can be tuned. This strategy could be an effective way to design different optical metasurfaces based on plasmonic nanohole array systems for applications such as ultra-thin optical filters, waveplates, polarizers, etc. For example, the 2RNH structure can serve as a polarization-modulated optical filter. When the incident light’s polarization is at *ϕ* = +45°, light with a wavelength of *λ*_a_ can pass through the device; when the polarization is changed to *ϕ* = −45°, light with a wavelength of *λ*_b_ can pass through the device. By alternately modulating a polarizer between *ϕ* = +45° and *ϕ* = −45°, light with wavelengths of *λ*_a_ and *λ*_b_ can pass through the device alternatively.

Experimentally, such a structure can be fabricated through advanced nanolithography methods, such as electron beam lithography, focus ion beam lithography, nanoimprinting, and others. Alternatively, the combination of nanosphere lithography and glancing angle deposition can also be employed [20]. Furthermore, the concept of coupling in compound nanohole structures can be extended to plasmonic structures designed for infrared (IR) or microwave applications. In these applications, the hole sizes would be significantly larger, and experimentally, the structures can be fabricated using conventional lithography methods or even printed board methods.

## Data Availability

All relevant data are within the paper.

## Supplementary Materials

The following supporting information can be downloaded at: https://www.mdpi.com/article/10.3390/nano13142135/s1, Figure S1: The original transmission spectra for Figure 2a; Figure S2: The original transmission spectra for Figure 3a; Figure S3: The original transmission spectra for Figure 5.
