# Supplementary material for: Plasmon Hybridizations in Compound Nanorod–Nanohole Arrays
Source: Nanomaterials (Basel). 2023 Jul 23;13(14):2135. doi: 10.3390/nano13142135 (PMC10383225; doi:10.3390/nano13142135)
Supplement: Supplementary file 1 [file nanomaterials-13-02135-s001.zip › nanomaterials-2499379-supplementary.pdf]

Supplementary Materials

Plasmon Hybridizations in Compound Nanorod–Nanohole Arrays

Shahab Razavi and Yiping Zhao

Department of Physics and Astronomy, University of Georgia, Athens, GA 30602, USA

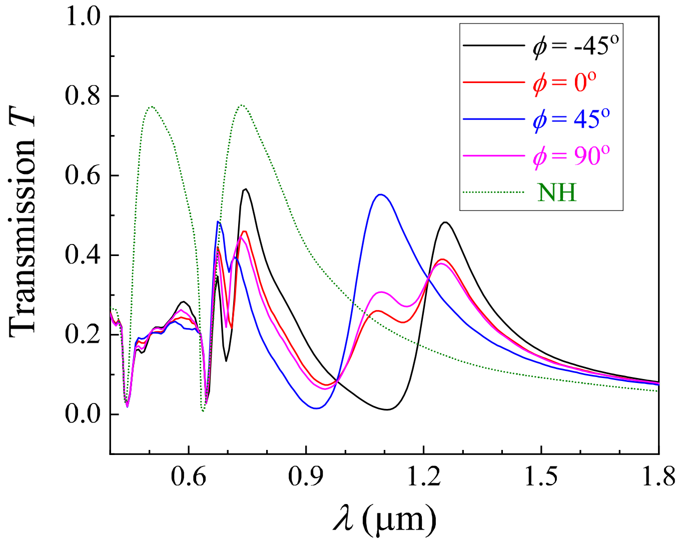

Figure S1. The original transmission spectra for **Figure 2a**.

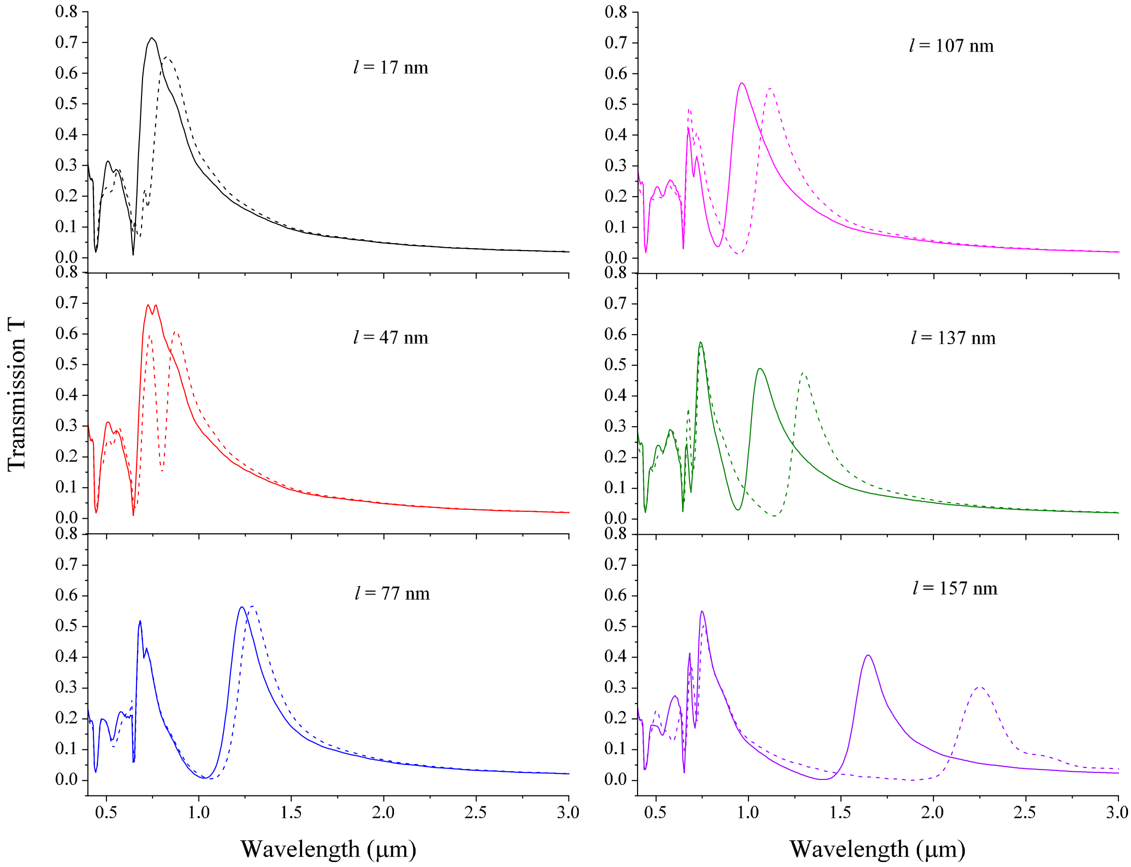

Figure S2. The original transmission spectra for **Figure 3a**.

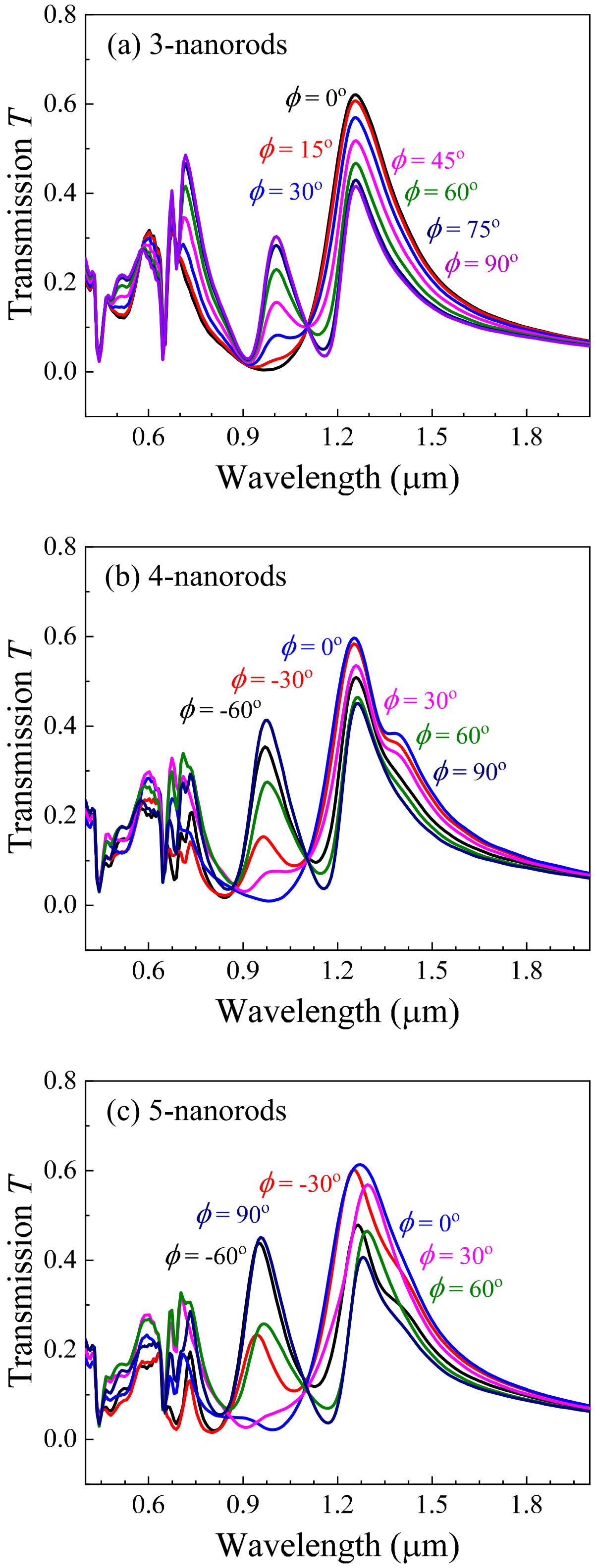

Figure S3. The original transmission spectra for **Figure 5**.
